# List of Amputations in the Pennsylvania Hospital, from March 1837, to March 1838, with the Results

**Published:** 1838-03-14

**Authors:** H. H. Smith

**Affiliations:** Res. Sur.


*List of Amputations in the Pennsylvania Hospital, from March* 1837, *to March* 1838, *with the results.*

(Reported by H. H. Smith, M. D., Res. Sur.)

*April* 8*th*,—Amputation of the thigh, for caries of the bones of the leg, (involving the knee joint,) and sloughing ulcer; died three days after. *Sept.* 11*th*,—Amputation of the arm, above the elbow, for a simple fracture of the end of the radius, rendered compound by a tight bandage, previous to his admission; union nearly perfect three weeks after, and a ring of bone from the humerus exfoliated during the cure—discharged cured, November 25th. *Sept.* 26*th*.—Amputation of the fore-arm, for caries of the wrist, ligatures remained till the end of the fourth week—discharged cured, 27th November. *Oct.* 28*th*.—Amputation of the fore-arm, for a gunshot wound of the hand, opening the wrist joint; discharged at his own request, November 30th—the stump healed a month after, considerable exfoliation of bone having taken place during the cure. *Nov.* 15*th*.—Amputation of the thigh, near the groin, for fungus hæmatodes, (reported at length in this number of the Examiner,) discharged January 10th, 1838. *Nov.* 17*th*.—Amputation of the shoulderjoint, for extensive laceration in machinery; reaction never complete, died three days after. *Jan.* 24*th*, 1838.—Amputation of the leg, below the knee, for caries of tarsal bones, (reported at length in No. 4,) cured Feb. 21st.

